# Conversion disorder in an 18-year-old boy: a case report

**DOI:** 10.1192/j.eurpsy.2022.1008

**Published:** 2022-09-01

**Authors:** C. Vilella Martín, P. García Vázquez, P. Fernández Perea, Y. Barrera García, A. Serrano García, J. De Santiago Sastre, R. Gómez Martínez, C. Franch Pato

**Affiliations:** Complejo Asistencial Universitario de León, Psychiatry, LEÓN, Spain

**Keywords:** conversive disorder

## Abstract

**Introduction:**

Conversion is the transformation of a conflict (unconscious) into a somatic symptom or a “non-verbal way of expressing psychological discomfort”, through somatizations. The disorder suggests a neurological or medical disease, associated psychological factors appear and is not produced intentionally.

**Objectives:**

To describe a case of conversion disorder.

**Methods:**

Retrospective review of clinical records and complementary test, including psychiatry, electrophysiology and neurology.

**Results:**

An 18-year-old boy came to the emergency room for paralysis. He has anesthesia of lower limbs. He shows indifference towards this symthoms.He denies any stressful situation. On examination, no psychotic or affective symptoms were observed. Belle indifference. Blood tests and a cranial CT scan were performed without alterations, so the patient was admitted for study. The electromyogram, lumbar puncture and cranial magnetic resonance show negative results. Suggestion is carried out, proving effective and recovering gait and sensitivity. These episodes are repeated up to 4 times until finally, during an interview with the family, episodes of bullying come to light. We work in therapy with a diagnosis of conversion disorder.

**Conclusions:**

The most frequent symptoms in conversion disorder are mutism, paralysis, anesthesia, blindness and seizures. It is usually monosymptomatic for each patient. Diferencial diagnosis with neurological pathology should be made.

**Disclosure:**

No significant relationships.

